# Failure of Less-Invasive Stabilization System (LISS) plating for periprosthetic distal femur fractures: Three case reports

**DOI:** 10.1097/MD.0000000000019195

**Published:** 2020-02-21

**Authors:** Zhen-Jiang Tian, Yan-Jie Liu, Bo-Jian Chen, Jun Wang, Cai-Li Niu, En-Hui Feng, Xiu-Jun Mai, Yong-Ming Huang

**Affiliations:** a Department of Orthopedic Surgery, Guangzhou University of Traditional Chinese Medicine, Guangzhou City; b Department of Orthopedic Surgery, The Second Affiliated Hospital of Guangzhou University of Traditional Chinese Medicine, Guangzhou,Guangdong Province, People's Republic of China.

**Keywords:** distal femur, failure, Less Invasive Stabilization System, periprosthetic fracture, total knee arthroplasty

## Abstract

**Rationale:**

Less-Invasive Stabilization System (LISS) plate is an internal fixation commonly used for the periprosthetic distal femur fractures. Failure associated with LISS plate has been rarely reported, and the reasons for LISS plate failure are multitudinous. Various advantages have been reported, but failures continue.

**Patient concerns:**

We present 3 cases illustrating the failure of Less-Invasive Stabilization System (LISS) plating for periprosthetic distal femur fractures. The shaft screws of the LISS plate broke in 2 cases, and the plate placement was incorrect in 1 case. Early weight bearing, obesity, osteoporosis, and lateral collateral ligament injury due to incorrect plate placement constituted the etiologies of LISS plate failure.

**Diagnosis:**

Failure of Less-Invasive Stabilization System (LISS) plating for periprosthetic distal femur fractures after Total knee arthroplasty.

**Interventions:**

Three patients underwent Less-Invasive Stabilization System plates removal with replacement of the total knee arthroplasty revision surgery with rotating hinged knee prosthesis.

**Outcomes:**

After completing the total knee arthroplasty revision surgery, all patients underwent regular follow-up examinations. Case 2 could walk unaided, without pain, final union was confirmed for both case 1 and case 3.

**Conclusion:**

Less-Invasive Stabilization System (LISS) plate provides satisfactory results in periprosthetic fractures after Total knee arthroplasty (TKA). The LISS plate has many advantages, but failures continue to occur. The causes for failure were early weight bearing, obesity, osteoporosis, and lateral collateral ligament (LCL) injury due to incorrect plate placement in our series. We recommend that protection or properly delay of weight-bearing, active anti-osteoporosis treatment, and intraoperative fluoroscopy are the effective methods to avoid failure.

## 1 Introduction

Periprosthetic fractures of the distal femur after total knee arthroplasty (TKA) are rare, with ranging range from 0.3 to 2.5%.^[1,2,4]^ However, due to extended lifespan, osteoporosis, and the increasingly elderly population, numbers of total knee replacement procedures are continually increasing yearly, and so are periprosthetic fractures.^[2]^ Treatment of such fractures is challenging because the fractures are around the femoral prosthesis. Furthermore, these patients are generally elderly with other underlying diseases.

Less-Invasive Stabilization System (LISS; Synthes, West Chester, PA) plates, have been placed percutaneously to attain maximal fixation and stability of complex fractures while minimizing soft-tissue dissection.^[3]^ The indirect reduction technique commonly used with LISS plates had been demonstrated good results in distal femoral fractures.^[1]^ The purpose of this paper is to analyze the reasons for LISS plate failure, and techniques that can be used to avoid failure.

## 2 Case presentation

### 2.1 Case 1

**Figure 1:**
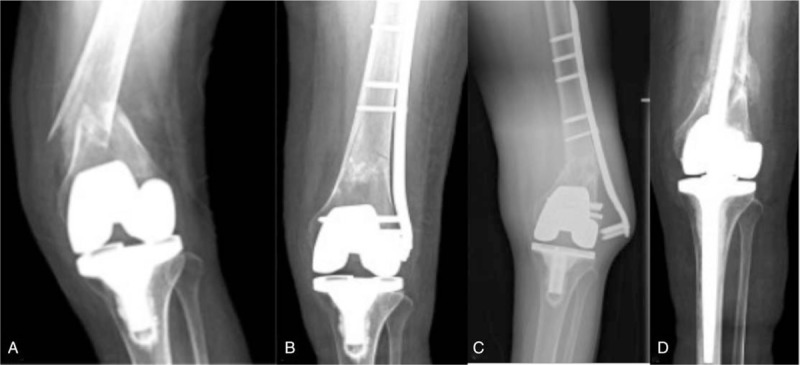
Anteroposterior radiographs of case 1, sustained a left Rorabeck type II closed distal femoral fracture (A), internal fixation of LISS plate was performed immediately (B). 7 months later, screws of LISS plate were broken and fracture displacement occurred again (C), underwent total knee arthroplasty revision surgery with rotating hinged knee prosthesis finally (D).

Case 1 was a 73-year-old female (Table 1) who had sustained a left Rorabeck type II closed distal femoral fracture after a fall from the stairs (Fig. 1A). Due to severe osteoarthritis of both knees, she underwent bilateral total knee arthroplasty surgeries 5 months prior. She was taken urgently to the operating room for periprosthetic fracture with a 9-hole LISS plate (Fig. 1B). Six screws the femoral condyle, and 4 screws were screwed in the proximal femur. Progressive weight bearing was not allowed until a significant callus was seen. Subsequently, she came to the hospital for anti-osteoporosis treatment and follow-up was performed monthly. Seven months later, 3 distal and 1 proximal screw of her LISS plate had broken; the side of the distal plate separated from the femoral condyle and could be seen clearly on X-ray (Fig. 1C). She underwent total knee arthroplasty revision surgery with rotating hinged knee prosthesis (Fig. 1D). Subcutaneous hematoma formed around the drainage tube 2 days later. Debridement was performed immediately under local anesthesia. Six months later, her fracture united, ambulated with 1 crutch, free of pain, with a knee range of motion (ROM) of 0° to 100^o^.

**Table 1:**
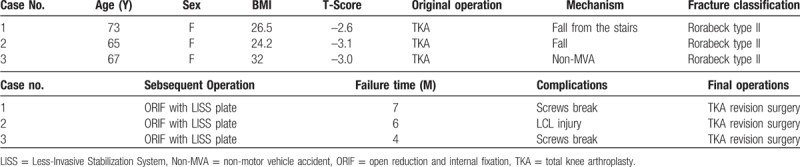
Patient data.

### 2.2 Case 2

**Figure 2:**
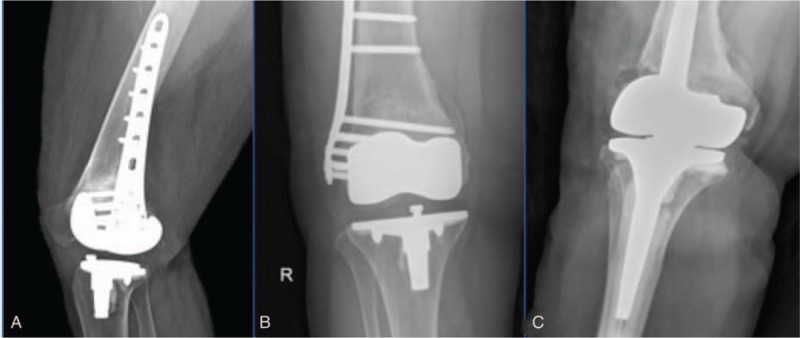
Anteroposterior and lateral radiographs of patient 2, the LISS plate was not centered on the femoral shaft (A), the gap of the lateral compartment was wider under varus stress (B), placement of the prosthesis was appropriate (C).

The second case referred from an outside institution was a 65-year-old female with a right Rorabeck type II fracture after a fall. She had a history of total knee arthroplasty surgery in both knees, as in Case 1. She underwent LISS plate fixation and significant callus formation was noted 6 months later (Fig. 2A). She was referred to our institution twice because of lateral knee pain after her internal fixation procedure, physiotherapy, and pharmacotherapy did not relieve her pain sufficiently. During the follow-up, her fracture was united, but the gap of the lateral compartment was wider than before on the postoperative x-rays, potentially generated potentially by partial or total injury of the lateral collateral ligament (Fig. 2B). She underwent LISS plate removal with replacement of the total knee arthroplasty (Fig. 2C). Six months after this procedure, she could walk unaided, without pain, but with partial numbness around the surgical incision.

### 2.3 Case 3

**Figure 3:**
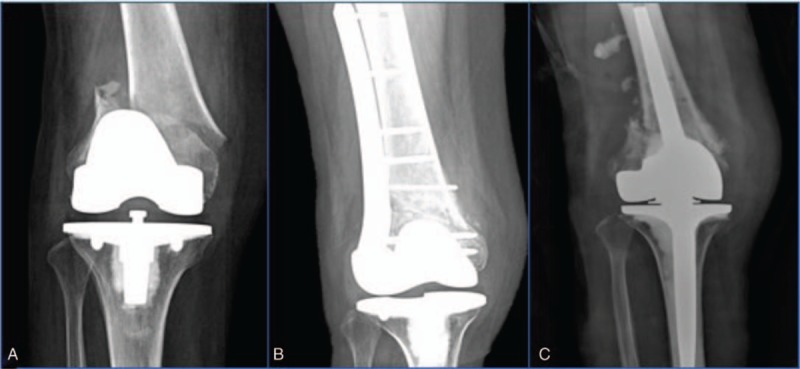
In case 3, the patient sustained a Rorabeck type II distal femoral fracture (A), bony callus was noted at the fracture site (B), total knee arthroplasty revision was beneficial for fracture healing.

The third case of plate failure occurred in a 67-year-old woman. Three distal screws were broken. Dislocation of the broken end was noted on her x-rays after a fall (Fig. 3). Just as with the other 2 patients, she underwent total knee replacement on account of the right knee osteoarthritis and sustained a Rorabeck type II distal femoral fracture in a non-motor vehicle accident. She was not noted to have a solid union after LISS plate fixation. Total knee arthroplasty revision surgery was performed the third day after the injury. At the last follow-up, she had no pain or limitations to activities of daily living activities. No further treatment was required. Final union was confirmed at 1 year after surgery for both case 1 and case 3.

## 3 Discussion

The rate increase of periprosthetic fractures is high every year, for reasons having to do both with aging of the general population and several comorbidities including osteoporosis and higher risk of falls, as well as the growing number of TKAs.^[4]^ The treatment of these fractures is difficult. Some variables should be considered, including general health status and functional demands, fracture location and morphology, bone quality, type of knee implant, and eventual loosening of prosthetic components. Acceptable alignments after fracture reduction are <5 mm translation, <5^o^ to 10^o^ angulation, <10^o^ rotational deformity, and <1 cm femoral shortening.^[5]^

We implanted 9 distal femoral LISS plates for periprosthetic fractures after TKA during the past 5 years. Two failed, and another was referred from an outside institution. According to Rorabeck,^[4–6]^ all of them were type II. The fractures were displaced and the implants were stable. The LISS, developed by AO in the late-1990s, offers several advantages for the fixation of distal femur fractures after TKA, including a limited soft-tissue dissection and periosteum and disruption of blood supply required for insertion. It is a fixed-angle device. It promotes rapid bone union with low risk of complications, such as hemorrhage and infection, compared with traditional techniques.^[5]^ Volumes of reports have emerged involving the beneficial results using LISS in stabilizing fractures around the knee.

In Case 1, due to the breakage of plate screws, the LISS plate missed the solid fixation, resulting in fracture displacement again. Although full weight-bearing was allowed at least 4 months after this procedure, she admitted that it was early. The literature on LISS plates is not specific concerning when weight bearing should be allowed, initial non-weight-bearing was required for between 8 and 12 weeks; toe-touch weight-bearing was stipulated for the first 4 to 6 weeks; partial weight bearing was initially required for the first 8 to 12 weeks following surgery.^[8]^ Fankhauser et al^[9]^ suggested that weight-bearing was determined on a case-by-case basis. Liu et al ^[7]^ recommended that weight-bearing should be deferred if the position of LISS plate was abnormal. Three distal and 1 proximal screws of the LISS plate were broken; a disadvantage of the LISS plating system is that the unicortical screws can result in pullout of the screw plate construct, especially in the thin cortex of osteoporotic bone, as a result of decreasing the working length of the locking screws.^[10]^ After the review of the present cases, we suggest the concept that the screw or plate could break because of the stress concentration effect at the plate end; the stress increases with adding distance from the fracture site, which generated by interaction between the LISS plate and screw. Further discussion will be pertained to the third case.

Case 2 was a case of postoperative pain after LISS plate fixation. A series of treatments did not work well. We found that the gap of the lateral compartment was wider on the x-ray, suggesting that her lateral collateral ligament of knee was injured. It was almost certainly due to incorrect plate placement. The LISS plate was not centered on the femoral shaft, according to the postoperative radiographs; the proximal end was placed anteriorly. Therefore, we suggested that fluoroscopic imaging during surgery is of paramount importance for assessing the position of the implant, and avoiding the LISS complications. The position of the implant was assessed by observing the shape of the holes in the implant, the direction of the locking head screws as well as relative position between the bone and the implant. Furthermore, the postoperative x-rays indicated that the gap of the lateral compartment was wider than before under varus stress, and slight internal rotation of the femoral component occurred. As the LISS is implanted through a small lateral approach without direct visualization of the LCL, the question has emerged as to whether damage to the LCL during LISS implantation might also be a possible cause for lateral knee pain.^[11]^ In a cadaver study, Freimoser et al reported that if the origin of the LCL was close to the implant, the LCL might be harmed by the locking screws of the plate; therefore, lateral knee pain, or lateral instability after implantation should be assessed in further treatment.^[12]^ Finally, it may affect the survival of the prosthesis. Consequently, intraoperative fluoroscopy should be used at each stage of the surgical procedure. Therefore, LCL injury should be considered as a potential cause, if a patient suffers from lateral knee pain or lateral knee instability after LISS procedure.

The third and first cases had several similarities. Bony callus was noted at the fracture site 4 months later. In both cases, the LISS plate screws of distal end were broken, confirmed during the final total knee arthroplasty revision surgery. The LISS failure in the Case 3 was almost certainly due to obesity and early, unsupervised weight-bearing. The body mass index (BMI) of the third patient was 32. Obesity is a prognostic risk factor of nonunion in distal femoral fractures treated with LLP or LISS, reported in a retrospective multicenter case-control study of 283 fractures.^[13]^ The screw breakages should be considered as a catastrophic complication after LISS application, as is screw pullout. The breakage of screws occurred in both the first and third cases, events that not been reported by other authors. In a previous study, some authors^[14]^ attributed screw pullout to the monocortical anchoring of the shaft screws. They supposed that regular bicortical screws could be used in order to obtain extra grip in the cortex, stimulated by the favorable results after bicortical screws had been used in the revision cases. However, we considered that the importance of this procedure is the correct position of the plate, rather than what types of locking screws had been used. Implant failure did not occur in any case with optimal implant positioning. Other controversies are how many shaft screws are needed, and length of LISS plate. Therefore, further research is required.

## 4 Conclusion

In conclusion, LISS plating provides satisfactory results in periprosthetic fractures after TKA. Nevertheless, the LISS plate has many advantages, and failed cases continue to occur. The causes for failure were early weight bearing, obesity, osteoporosis, and LCL injury due to incorrect plate placement in our series. We firmly believe that the ways to avoid failure are as follows: protection or properly delay of weight-bearing for longer periods of time, active anti-osteoporosis treatment, and intraoperative fluoroscopy should be used to make sure the plate is placed in the optimal position.

## Acknowledgments

We would like to thank American Journal Experts for English language editing.

## Author contributions

**Data curation:** Jun Wang.

**Investigation:** Yan-jie Liu.

**Methodology:** Bo-jian Chen, Cai-li Niu.

**Supervision:** En-hui Feng, Xiu-jun Mai.

**Writing – original draft:** Zhen-jiang Tian.

**Writing – review & editing:** Yong-ming Huang.
